# Temporal trends in lupus pregnancy over four decades in a referral centre: pregnancy planning and hydroxychloroquine use are associated with improved outcomes

**DOI:** 10.1093/rap/rkaf137

**Published:** 2025-12-04

**Authors:** Dionysia Mandilara, Spyridon Katechis, Sofia Flouda, Katerina Chavatza, Konstantinos Drougkas, Dimitrios Katsifis-Nezis, Dimitrios T Boumpas, Antonis Fanouriakis

**Affiliations:** Rheumatology and Clinical Immunology Unit, Attikon University Hospital, Medical School, National and Kapodistrian University of Athens, Athens, Greece; Rheumatology and Clinical Immunology Unit, Attikon University Hospital, Medical School, National and Kapodistrian University of Athens, Athens, Greece; Department of Rheumatology, Asklepieion General Hospital, Athens, Greece; Rheumatology and Clinical Immunology Unit, Attikon University Hospital, Medical School, National and Kapodistrian University of Athens, Athens, Greece; Rheumatology and Clinical Immunology Unit, Attikon University Hospital, Medical School, National and Kapodistrian University of Athens, Athens, Greece; Rheumatology and Clinical Immunology Unit, Attikon University Hospital, Medical School, National and Kapodistrian University of Athens, Athens, Greece; Rheumatology and Clinical Immunology Unit, Attikon University Hospital, Medical School, National and Kapodistrian University of Athens, Athens, Greece; Rheumatology and Clinical Immunology Unit, Attikon University Hospital, Medical School, National and Kapodistrian University of Athens, Athens, Greece; Rheumatology and Clinical Immunology Unit, Attikon University Hospital, Medical School, National and Kapodistrian University of Athens, Athens, Greece

**Keywords:** systemic lupus erythematosus, preconception counselling, planned pregnancy, hydroxychloroquine, temporal trends

## Abstract

**Objective:**

To evaluate temporal trends in pregnancy management and outcomes in women with SLE and explore the impact of pregnancy planning on maternal and foetal complications.

**Methods:**

We conducted a retrospective study including women with SLE with one or more pregnancies after diagnosis or diagnosed during pregnancy. Data were collected through questionnaires and medical records. To assess temporal trends, the study period (1985–2024) was divided into five intervals with similar pregnancy and patient distribution. Using generalized estimating equations, we identified risk factors for adverse pregnancy outcomes and evaluated temporal trends in pregnancy characteristics.

**Results:**

We recorded 109 pregnancies from 65 women; 70.6% resulted in live births. Planned pregnancies were associated with a lower flare risk [odds ratio (OR) 0.31 (95% CI 0.11, 0.89)] and a higher likelihood of live birth [OR 3.31 (95% CI 1.41, 7.8)]. HCQ use during pregnancy was independently associated with a reduced flare risk [OR 0.26 (95% CI 0.08, 0.8)]. Pregnancies before 2005 were less often planned [OR 0.10 (95% CI 0.03, 0.4)], while HCQ use during pregnancy increased over time [OR 0.56 (95% CI 0.34, 0.95), for the period 1985–2005 *vs* 2021–2024], with discontinuation decreasing from 33% to <2%. The predicted probability of preventive aspirin use in patients without antiphospholipid syndrome increased from 12% before 2005 to 46% in 2021–2024, whereas continuous glucocorticoid use decreased from 58% before 2005 to 16% after 2020. The probability of foetal complications showed a decreasing trend after 2016.

**Conclusion:**

Pregnancy management and outcomes improved over time in SLE; planned pregnancy and HCQ use were associated with favourable results.

Key messages

- Planned pregnancies and hydroxychloroquine use were independently associated with improved pregnancy outcomes in lupus.
- Temporal trends demonstrated increased pregnancy planning and treatment adherence over recent decades.
- Refining pregnancy planning definitions and developing targeted counselling interventions are essential to optimize pregnancy outcomes.

## Introduction

Pregnancy in patients with SLE presents significant challenges [1, 2]. Despite considerable progress in disease management and a trend towards improved maternal and foetal outcomes, women with SLE still experience higher rates of both maternal and foetal complications compared with the general population [3]. Maternal risks include disease flare, pre-eclampsia and increased rates of caesarean section (CS), while higher rates of foetal loss, prematurity and small for gestational age (SGA) infants are also reported in SLE pregnancies [4, 5]. As a result, many women with lupus hesitate to pursue pregnancy and often have smaller family sizes [6].

A number of studies have investigated factors contributing to the increased risk of adverse pregnancy outcomes in women with lupus [2, 7–9]. High disease activity 6–12 months prior to conception, a history of three or more flares during the preceding year, hypocomplementemia, a history of LN, discontinuation of HCQ and the presence of anti-Ro and aPL antibodies are among the major risk factors for disease flare during pregnancy and poor pregnancy outcomes [10]. However, the reported rates of maternal and foetal complications, including disease flares, vary considerably across studies. This variation often reflects differences in inclusion criteria and definitions used for complications [11, 12].

In contrast to the extensive literature on clinical outcomes, only a few small-scale studies have explored the level of awareness among women with SLE regarding the importance of pregnancy planning and its potential impact on maternal and foetal outcomes [13, 14]. Recent research has focused on the unmet informational needs of patients with autoimmune rheumatic diseases [7, 15, 16]. However, what constitutes a ‘planned pregnancy’ is not well-defined, which contributes to inconsistencies in data interpretation and clinical guidance.

Based on the above, we undertook a single-centre retrospective study in a population of Greek women with SLE who either conceived at least once after the diagnosis of the disease or were diagnosed during pregnancy. We aimed to investigate risk factors associated with adverse pregnancy outcomes, with a particular emphasis on the role of pregnancy planning, and temporal trends in pregnancy management and outcomes over the years.

## Methods

### Study population and data collection

This was a retrospective study, including women with SLE who fulfilled the 2012 SLICC [17] and are currently followed at the Rheumatology and Clinical Immunology Unit of ‘Attikon’ University Hospital, Athens, Greece. Eligible participants had either conceived at least once after SLE diagnosis or were diagnosed with SLE during pregnancy, regardless of pregnancy outcomes. For women who had more than one pregnancy following the diagnosis of SLE, all pregnancies were included in the analysis.

Data were collected through both printed and electronic questionnaires, as well as by reviewing the patients’ medical records. The content of the questionnaires was identical in both formats and the researchers’ involvement remained consistent across the two methods. Questionnaires were distributed via e-mail to patients with an available e-mail address, while others received them in person during regular outpatient visits. The review of medical records served to confirm the collected data and supplement them with additional clinical information, when necessary. Participation in the study was entirely voluntary and written informed consent was obtained from all patients. The study was approved by the Scientific Council and the Ethics and Bioethics Committee of ‘Attikon’ University Hospital (protocol 657/10-09-2024). Data collection and handling complied with the principles of the Declaration of Helsinki.

Collected data included patient demographic characteristics and medical history, including both initial and cumulative manifestations during follow-up. Prior to pregnancy, disease was characterized as severe if there was a history of major organ involvement or disease manifestations that required treatment with potent immunosuppressive agents such as CYC or rituximab (RTX). Obstetric history was also recorded, with particular emphasis on the following characteristics: whether patients had received prior counselling regarding the importance of pregnancy planning, whether pregnancies were planned or unplanned (see definition below), mode of conception and delivery, gestational age at birth, birth weight, maternal and foetal complications, medications administered during the preconception period and throughout pregnancy and changes in disease activity during gestation. Continuous glucocorticoid (GC) use was defined as GC administration throughout all trimesters of pregnancy, regardless of the indication. GC use for non-flare indications referred to GC administration at any point during pregnancy without evidence of a flare. The immunological profile was documented, focusing on the presence of anti-Ro and aPL antibodies.

### Adverse foetal and maternal outcomes

Respective definitions are presented in Supplementary Tables S1 and S2 [18–26], available at *Rheumatology Advances in Practice* online.

### Disease activity during pregnancy

Based on the course of disease activity, pregnancies were classified into three groups: pregnancies in which at least one flare occurred, pregnancies in which the disease went into remission and pregnancies where disease activity was either absent or low before pregnancy and remained stable throughout its course. A disease flare was defined following the definition established by the international consensus for a definition of disease flare in lupus [27, 28], i.e. appearance or worsening of specific SLE-related symptoms such as cutaneous, articular, neurological, cardiopulmonary or renal symptoms and cytopenias not attributed to other causes. Active LN was characterized by the presence of proteinuria >0.5 g/24 h and/or active urinary sediment (such as more than three red blood cells or more than five white blood cells per high-power field or the presence of cellular casts) [27]. Severe disease flares were defined as those characterized by major organ involvement or requiring the administration of immunosuppressive agents such as CYC or RTX. Remission was defined according to the definition of remission in SLE (DORIS) criteria (clinical SLEDAI-2K = 0, Physician’s Global Assessment (PGA) <0.5, prednisone dose ≤5 mg/day, stable dose of antimalarials, immunosuppressants and biologics) [29].

### Definition of a planned pregnancy

Planned pregnancy was defined as a scheduled pregnancy, which occurred following specific consultation with the treating rheumatologist, and required the disease to be in remission for at least 6 months and the medication regimen to include drugs compatible with pregnancy.

### Statistical analysis

For continuous variables, median and interquartile range (IQR) or mean (s.d.) values are presented, as appropriate, while frequencies are shown for categorical variables. Categorical data between groups were compared using the chi-squared test or Fisher’s exact test, as appropriate. To identify risk factors associated with the occurrence of flares during pregnancy and foetal complications (dependent variables), generalized estimating equations (GEEs) were employed, as multiple pregnancies per patient were included and the observations were not independent. Specifically, a binomial logit model with an unstructured correlation matrix was applied. Potential risk factors were first evaluated through univariable analysis and variables with a *P*-value <0.2 were included in the multivariable model, using a stepwise backward selection method. To explore potential temporal trends in pregnancy-related parameters, especially pregnancy planning, pregnancies were categorized into five calendar periods, each comprising a roughly equal number of pregnancies or patients, based on the year of conception: group 1 (1985–2005), group 2 (2006–2014), group 3 (2015–2016), group 4 (2017–2020) and group 5 (2021–2024). The predicted probability for each parameter was assessed across these time periods, using the most recent group as the reference category. A two-sided *P*-value <0.05 was considered statistically significant for all analyses. All data were recorded and analysed using SPSS Statistics version 29.0.2.0 (IBM, Armonk, NY, USA).

## Results

### Patient and pregnancy characteristics

A total of 65 Caucasian SLE patients with a total of 109 pregnancies were included in the study. Patients’ demographic and clinical characteristics are presented in Supplementary Table S3, available at *Rheumatology Advances in Practice* online. In total, 77 live births (70.6%) were recorded, including 6 twin gestations. Pregnancy characteristics are presented in Table 1. Foetal and maternal complications were observed in 67% (*n* = 73) and 41.3% (*n* = 45) of pregnancies, respectively (Table 2). Preterm birth was the most common adverse foetal outcome; however, it should be noted that 33% (*n* = 10) of elective CSs were performed at the end of the 36th week of gestation, suggesting that a proportion of prematurity was iatrogenic, resulting from the obstetricians’ decision to terminate pregnancies earlier in women with SLE. The incidence rates of individual maternal complications were generally low, with disease flare during pregnancy being the most common [24.8% (*n* = 27)]. During the majority of pregnancies [73.4% (*n* = 80)], the disease remained stable, while remission was observed in 2 cases (1.8%). Diagnosis of SLE during pregnancy was established in six patients. Most flares were mild [63% (*n* = 17)] and primarily involved the skin [74.1% (*n* = 20)] and/or joints [66.7% (*n* = 18)]. Kidney involvement was noted in eight pregnancies, in five of which LN was first diagnosed during pregnancy.

**Table 1:** Pregnancy characteristics in women with SLE.

Pregnancy characteristics^a^	Values
Characteristics assessed per pregnancy (*n* = 109)	
Maternal age at conception, years, median (IQR)	32 (6.8)
Pregnancies, *n* (%)	109
Live births	77 (70.6)
Foetal losses	32 (29.4)
Major organ involvement prior to conception, *n* (%)	27 (24.8)
Renal involvement	20 (18.3)
CNS involvement	7 (6.4)
Smoking at the time of conception, *n* (%)	29 (26.6)
Time to conception, *n* (%)	
<12 months	98 (89.9)
>12 months	11(10.1)
Medication before conception, *n* (%)	
HCQ	84 (77.1)
GCs	53 (48.6)
LDA	15 (13.8)
LMWH	9 (8.3)
None	19 (17.4)
Medication during pregnancy, *n* (%)	
HCQ	72 (66.1)
MTX	1 (0.9)
AZA	23 (21.1)
CSA	2 (1.8)
GCs	57 (52.3)
LDA	38 (34.9)
LMWH	31 (28.4)
IVIG	3 (2.8)
None	21 (19.3)
Autoantibodies [assessed per patient (*n* = 65) at the time of conception], *n* (%)	
ANA	65 (100)
Anti-dsDNA	29 (44.6)
Anti-Ro	19 (29.2)
Anti-La	2 (3.1)
Anti-CL	10 (15.4)
LAC	5 (7.7)
Anti-β2GP1	11 (16.9)

a Data include demographic, clinical, immunological and treatment characteristics prior to and during pregnancy. ‘Time to conception’ reflects the interval from pregnancy attempt to confirmed conception. Autoantibody status was assessed per patient at the time of conception and remained constant across pregnancies.

anti-dsDNA: anti-double-stranded DNA antibodies; anti-CL: anti-cardiolipin antibodies; LAC: lupus anticoagulant; anti-β2GP1: anti-β2 glycoprotein I antibodies.

**Table 2:** Foetal and maternal outcomes.

Outcomes	Values
Newborns, *n* (%)^a^	83
Female	53 (63.9)
Male	30 (36.1)
Mode of delivery, *n* (%)^b^	
Vaginal delivery	29 (37.7)
Elective CS	30 (39)
Emergency CS	18 (23.3)
Week of gestation at delivery, *n* (%)^b^	
28–33 weeks	7 (9.1)
34–36 weeks	26 (33.8)
>37 weeks	44 (57.1)
Week of foetal loss, *n* (%)^c^	
<10 weeks	22 (68.75)
10–20 weeks	6 (18.75)
>20 weeks	4 (12.5)
Birth weight, g, mean (s.d)^a^	2600 (840)
Low birth weight, *n* (%)^a^	34 (41)
Foetal complications, *n* (%)]^d^	73 (67)
Foetal loss^d^	32 (29.4)
Neonatal death^d^	0
SGA^a^	16 (19.28)
IUGR^a^	13 (15.7)
Preterm birth^b^	33 (42.9)
Neonatal lupus, *n* (%)^d^	
Cutaneous neonatal lupus	1 (0.9)
Complete AV block	2 (1.8)
Maternal complications, *n* (%)^d^	45 (41.3)
Gestational hypertension	3 (2.8)
Gestational diabetes	3 (2.8)
Thrombosis	1 (0.9)
Infection	4 (3.7)
Pre-eclampsia	3 (2.8)
Eclampsia	0
HELLP syndrome	0
Increased uterine artery resistance	1 (0.9)
Placental abruption	4 (3.7)
PROM	3 (2.8)
Vaginal bleeding	3 (2.8)
Oligohydramnios	3 (2.8)
Polyhydramnios	1 (0.9)
Gestational osteoporosis	1 (0.9)
Hyperemesis gravidarum	6 (5.5)
Disease flares	27 (24.8)

AV block: atrioventricular block; PROM: premature rupture of membranes; HELLP: haemolysis, elevated liver enzymes, low platelets.

a Mean (SD) and percentages were calculated based on the total number of newborns (*n* = 83).

b Percentages were calculated based on the total number of live births (*n* = 77).

c Percentages were calculated based on the total number of foetal losses (*n* = 32).

d Percentages were calculated based on the total number of pregnancies (*n* = 109).

### Temporal trends in pregnancy planning and management practices in women with SLE

#### Pregnancy planning

**Figure 1.**
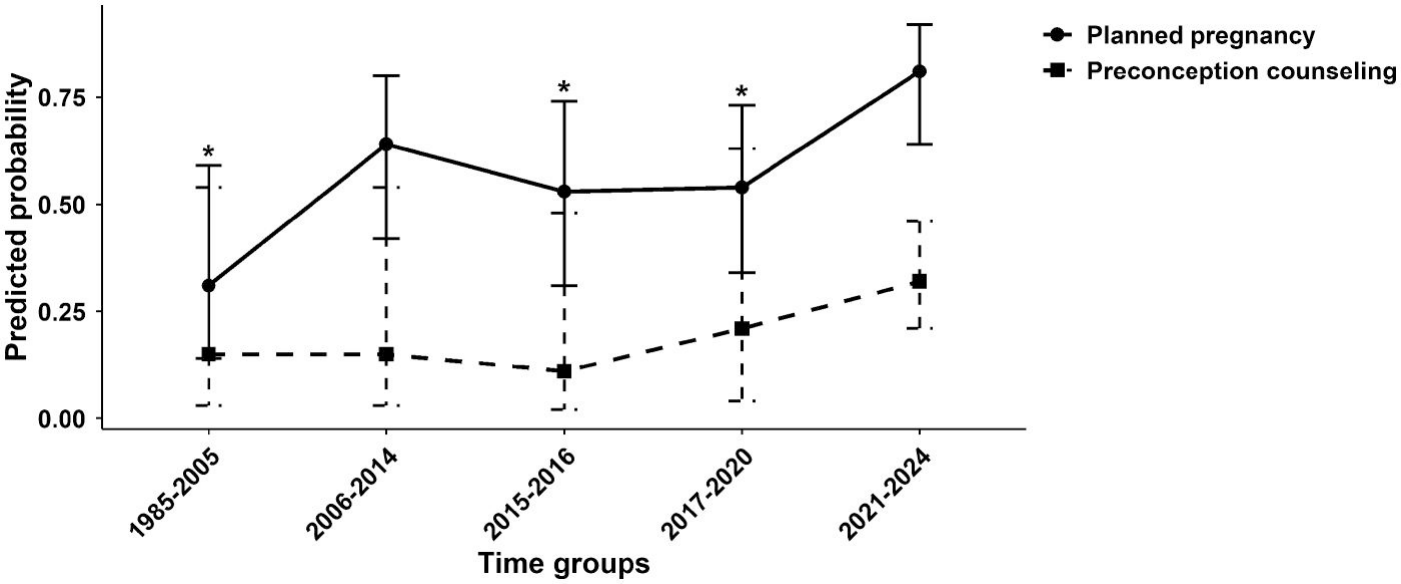
Temporal trends in the predicted probability of pregnancy planning and preconception counselling in lupus pregnancies. Predicted probabilities of pregnancy planning and preconception counselling across five sequential conception year groups (1985–2024), estimated using GEEs. Plots represent estimated marginal means ± 95% CIs. *P < 0.05 compared with group 5 (2021–2024)

Of the total number of pregnancies, 46.8% (*n* = 51) were considered planned. Notably, pregnancies in earlier time periods were significantly less likely to be planned compared with more recent years, with the oldest time group showing nearly a 90% lower likelihood of planned pregnancy [odds ratio (OR) 0.1 (95% CI 0.03, 0.4)] (Fig. 1; Supplementary Table S4, available at *Rheumatology Advances in Practice* online). Although a statistically significant linear association between the year of conception and the likelihood of receiving family counselling by a healthcare professional (*P* = 0.67) was not found, possibly due to the small sample size, a trend towards increased counselling in more recent years was evident (Fig. 1; Supplementary Table S4, available at *Rheumatology Advances in Practice* online).

#### HCQ use

**Figure 2.**
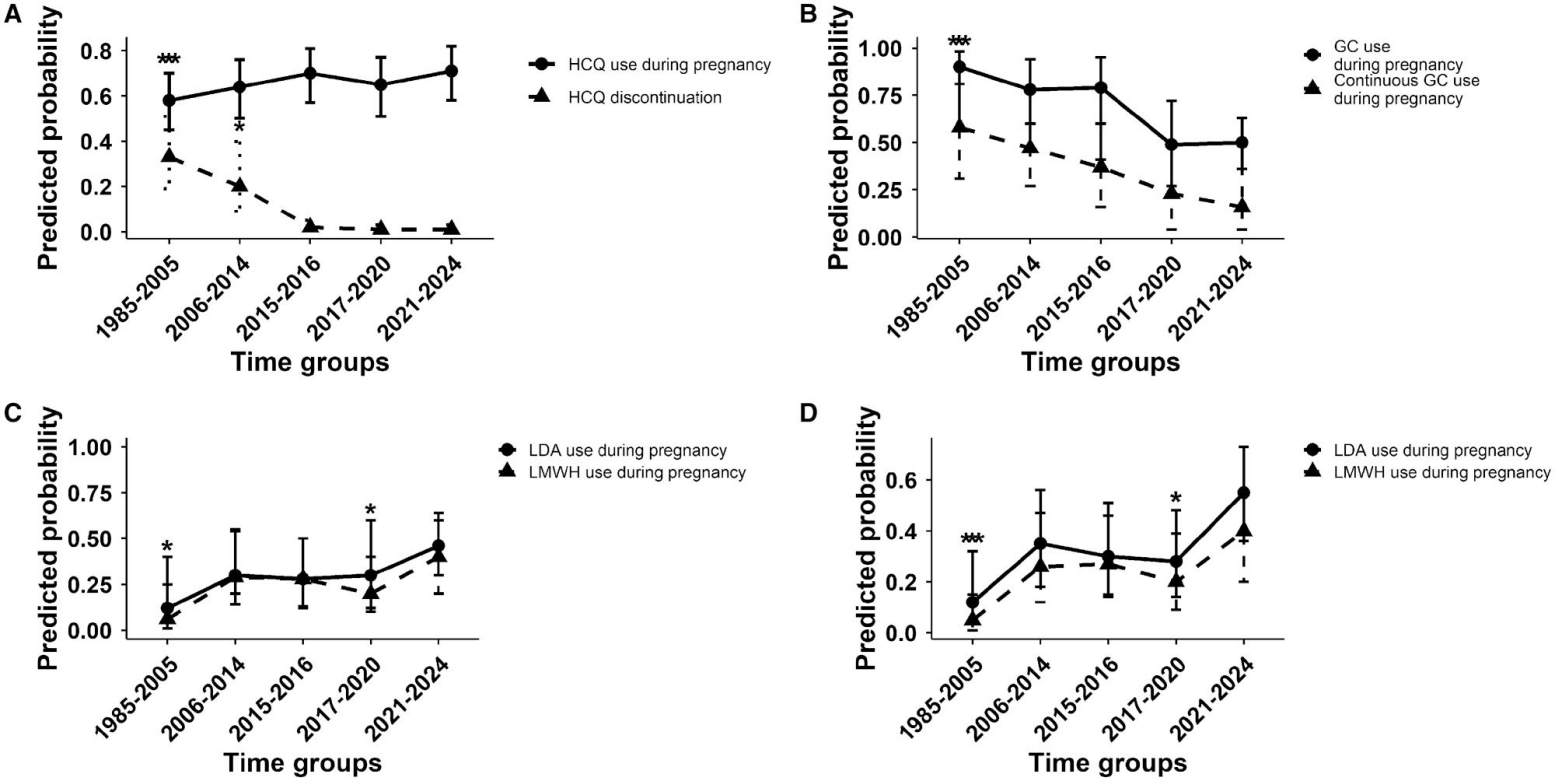
Temporal trends in medication use during lupus pregnancies. Predicted probabilities of medication use in lupus pregnancies across five time groups (1985–2024), estimated using GEEs. Plots show estimated marginal means ± 95% CIs. *P < 0.05 compared with 2021–2024 (group 5), indicating significance only for the variable above whose error bar it is placed. **P < 0.05 compared with group 5, indicating significance for both variables. **(A)** HCQ use and discontinuation. **(B)** GC use for non-flare indications and continuous GC use. **(C)** LDA and LMWH in patients without APS. **(D)** LDA and LMWH in patients without APS/major organ involvement

Patients in the earliest time period were significantly less likely to receive HCQ during pregnancy compared with those in the most recent period [OR 0.56 (95% CI 0.34, 0.95)]. No statistically significant differences were noted for the intermediate periods, although a progressive increase in HCQ use over time was seen (Fig. 2; Supplementary Table S4, available at *Rheumatology Advances in Practice* online). Furthermore, among patients who were already receiving HCQ prior to conception, the likelihood of discontinuing treatment during pregnancy varied across time. Predicted probabilities of discontinuation were highest in earlier years, ≈33% in the earliest group [predicted probability 0.33 (95% CI 0.19, 0.51)] and 20% in the second oldest quintile [predicted probability 0.2 (95% CI 0.09, 0.4)], while they declined markedly in later years, ranging between 1% and 2% across the three most recent quintiles [predicted probability in group 5 0.01 (95% CI 0.009, 0.03)] (Fig. 2). Notably, among pregnancies that occurred within the last decade (2015–2024), no patients discontinued HCQ, further supporting the trend toward its consistent use during gestation in women with SLE.

#### GC use

In the total cohort of pregnancies, the predicted probability of GC use for non-flare indications at any point during gestation declined over time, particularly after 2016, with stabilization in recent years. The probability decreased from 90% before 2005 to 79% in 2015–2016 and to 50% after 2020. Among pregnancies in which GCs were administered (*n* = 57), the predicted probability of continuous GC use showed a similar temporal trend, declining from 58% before 2005 to 37% in 2015–2016 and to 16% after 2020 (Fig. 2; Supplementary Table S4, available at *Rheumatology Advances in Practice* online).

#### Antiplatelet/anticoagulant use

Overall, low-dose aspirin (LDA) was administered in 38 pregnancies (8 with APS and 30 prescribed by gynaecologists as high-risk pregnancies) and low-molecular-weight heparin (LMWH) in 31 (9 with APS, 22 prescribed by gynaecologists as high-risk pregnancies). Among pregnancies without APS, preventive LDA use increased markedly overall, including in patients without major organ involvement (e.g. LN), whereas LMWH use rose less substantially (Fig. 2; Supplementary Table S4, available at *Rheumatology Advances in Practice* online).

### Predictors and temporal trends of adverse outcomes during pregnancy

Regarding disease flare during pregnancy, GC use and aPL antibodies were independent risk factors for flare occurrence (Table 3). Conversely, planned pregnancy and the use of HCQ during gestation were identified as independent protective factors. Specifically, patients taking HCQ during pregnancy and patients with planned pregnancy had a 74% and 69% lower likelihood, respectively, of experiencing a flare (Table 3). Smoking at the time of conception and a history of LN were identified as independent predictors of adverse foetal outcomes (Table 3).

**Table 3:** Predictors of pregnancy outcomes in women with SLE.

Variables^a^	Disease flares^a^		Adverse foetal outcomes^a^		Live births^a^	
	Univariable analysis, OR (95% CI)^a^^,^^b^	Multivariable analysis, OR (95% CI)^a^^,^^b^	Univariable analysis, OR (95% CI)^a^^,^^b^	Multivariable analysis, OR (95% CI)^a^^,^^b^	Univariable analysis, OR (95% CI)^a^^,^^b^	Multivariable analysis, OR (95% CI)^a^^,^^b^
Age at conception	0.98 (0.9–1.1)		1.02 (0.93–1.1)		0.9 (0.9–1.09)	
Number of pregnancies (1st–3rd)	1.58 (0.37–6.74)		0.74 (0.43–1.24)		1.31 (0.75–2.28)	
Multiple pregnancy	0.99 (0.17–6.01)		2.88 (0.4–20.72)		1.28 (0.24–6.8)	
Smoking at conception	1.02 (0.33–3.19)		**2.93 (0.97–8.82)**	4.81 (1.48–15.67)*	**0.29 (0.12–0.67)** *	0.25 (0.1–0.63)*
History of any complications in previous pregnancy	1.28 (0.46–3.56)		**2.04 (0.86–8.56)**	2.36 (0.86–6.5)	1.1 (0.45–2.7)	
History of LN	1.28 (0.35–4.7)		**3.03 (0.86–10.7)**	3.9 (1.12–13.7)*	ΝΑ	
History of major organ involvement	1.2 (0.35–4.13)		2.1 (0.67–6.53)		**0.29 (0.18–0.48)** *	0.18 (0.07–0.43)*
aPL antibodies	**3.9 (0.93–16.5)**	6.75 (1.23–36.8)*	3.31 (0.36–30.7)		0.48(0.09–2.63)	
Anti-Ro antibodies	NA		0.85 (0.32–2.24)		1.28 (0.48–3.4)	
Planned pregnancy	**0.54 (0.22–1.3)**	0.31 (0.11–0.89)*	0.94 (0.42–2.09)		**5.54 (2.84–10.84)** *	3.31 (1.41–7.8)*
Use of HCQ during pregnancy	**0.47 (0.17–1.3)**	0.26 (0.08–0.8)*	1.13 (0.46–2.79)		0.81 (0.36–1.87)	
Use of LDA during pregnancy	0.7 (0.25–1.95)		1.48 (0.51–4.27)		1.26 (0.46–3.5)	
Use of LMWH during pregnancy	1.64(0.61–4.33)		1.6 (0.59–4.17)		1.21 (0.47–3.11)	
Use of GCs during pregnancy	**5.3 (1.46–19.3)** *	8.24 (2.72–24.94)*	**2.06 (0.83–5.14)**	1.81 (0.63– 5.2)	0.8 (0.37–17.3)	
Disease flare during pregnancy	NA		1.55 (0.68–3.5)		0.62 (0.25–1.59)	

NA: not applicable.

a Univariable and multivariable analyses using GEEs were performed to examine the association between demographic, clinical and immunological variables and three pregnancy outcomes: disease flares, adverse foetal outcomes and live birth.

b Values are presented as ORs with 95% CIs. Bold values in the univariable analysis indicate variables with *P* < 0.2, which were subsequently considered for inclusion in the multivariable model.

* Statistically significant associations (*P* < 0.05).

Regarding the likelihood of live birth, smoking at the time of conception was found to be an independent risk factor for foetal loss, as was a history of major organ involvement due to the disease, with the latter being associated with an 82% lower likelihood of live birth. Notably, pregnancy planning emerged as a significant protective factor, with patients who had planned their pregnancies having a 3.3-fold higher likelihood of achieving a live birth.

Regarding foetal complications, a decrease in the proportion of pregnancies experiencing any complication was observed in the most recent time groups (Supplementary Fig. S1; Supplementary Table S4, available at *Rheumatology Advances in Practice* online). However, among pregnancies that were affected, the likelihood of having multiple concurrent complications was higher in recent time groups. This indicates that although fewer pregnancies overall had complications, the burden of complications per affected pregnancy tended to be greater. This observation may reflect the increased prevalence of pregnancies in women with a history of major organ involvement over time, especially LN, factors associated in our study with a higher risk of foetal complications (Table 3; Supplementary Table S5, available at *Rheumatology Advances in Practice* online). Additionally, maternal age at conception, a recognized risk factor, was significantly higher in later groups [30] (Supplementary Table S5, available at *Rheumatology Advances in Practice* online). Regarding individual foetal complications, trends were difficult to assess due to the relatively small number of cases per period. No clear pattern was observed for foetal loss, preterm birth or SGA, reflecting fluctuations rather than consistent changes over time. In contrast, the probability of intrauterine growth restriction (IUGR) increased, possibly reflecting changes in diagnostic criteria of this condition rather than a true increase in incidence (Supplementary Fig. S1, available at *Rheumatology Advances in Practice* online).

## Discussion

We conducted a retrospective study including 109 pregnancies in women with SLE, adopting a broader perspective than previous research, which focused mainly on adverse outcomes [5, 11, 31–34]. Our study also explored women’s awareness of family planning, the effect of the latter on maternal and foetal outcomes, as well as temporal trends in pregnancy management and outcomes. We found that planned pregnancies were associated with fewer disease flares and less frequent foetal loss, while HCQ use was linked to reduced flare risk.

Although the importance of reproductive counselling in rheumatic diseases has been long recognized [35], prior to the 2016 EULAR recommendations [10] only a few studies [6, 36] had emphasized preconception counselling and pregnancy planning. At the same time, while a few studies supported the safety and potential benefits of continuing HCQ during pregnancy, including a double-blind randomized trial [37, 38], most of them were based on small patient cohorts. This is consistent with our early time periods (groups 1–3), where preconception counselling was infrequent, HCQ was often discontinued in pregnancy (groups 1–2) and GCs were commonly maintained throughout pregnancy, reflecting the limited evidence and lack of formal guidance available at that time. The study by Clowse *et al.* [39] had the greatest impact on HCQ use, and by the mid-2010s (group 3), accumulating evidence supporting the safety and benefits of HCQ in pregnancy led to a notable shift towards its sustained use. Among the few studies that have assessed temporal trends in HCQ use during pregnancy, Bermas *et al.* [40] reported an increased use of HCQ in 2015 compared with 2001, while Barnardo *et al.* [41] observed an increasing, though not substantial trend, with a marked increase in 2015 (70%). A similar trend was also identified in our cohort. Following the release of the EULAR and British Society for Rheumatology (BSR) recommendations in 2016 [10, 42], further improvements became evident in groups 4 and 5 of our study, with increased rates of preconception counselling and greater adherence to guideline-supported therapies. Finally, the 2020 ACR reproductive health guidelines [43] likely contributed to the continuation of these positive trends, including broader aspirin use among women without APS and LN.

Over the 4 decades covered by our study, the proportion of pregnancies affected by foetal complications declined in most recent groups. Interestingly though, among complicated pregnancies, multiple concurrent adverse outcomes appeared more frequently in later periods; this may suggest that women entering pregnancy nowadays often comprise a higher-risk subgroup than before, as they are frequently older and with more serious disease or obstetric comorbidities. Improvements in pregnancy management likely contributed to the overall reduction in complications, although those who remain at risk tend to constitute more complex cases. The presence of aPL antibodies, LN and organ damage remained strong predictors of disease flares and pregnancy complications. These non-modifiable risk factors further underline the importance of pregnancy planning and adherence to recommended treatments in this high-risk population.

Beyond advances in rheumatologic care, developments in obstetric practice have likely influenced these favourable trends. Until the early 2000s, ultrasonography was limited in availability and image quality [44] and IUGR definitions relied primarily on umbilical artery Doppler, potentially missing milder cases. The increased research and use of new indices for detecting late-onset IUGR after 2005 (group 2) [45] are likely related to the upward trend observed in our study rather than a true increase in incidence.

Antenatal care in the general population of pregnant women has also evolved over the years [46–48]. In Greece, the absence of a respective national registry for antenatal quality indicators limits precise information on visit frequency across study periods [48]. Nevertheless, available data suggest high access to prenatal monitoring, with one of the highest frequencies of obstetric visits (six to eight visits) and ultrasound examinations in Europe (more than seven). Moreover, follow-up during the SARS-CoV-2 pandemic (groups 4–5) remained stable [48], in contrast with other European countries. Despite Greece having the highest prematurity and CS rates in Europe [49, 50], preterm births in our cohort showed no clear temporal trend. One-third of elective CSs occurred at 36 weeks, highlighting the need to distinguish spontaneous from iatrogenic preterm births in future studies.

The higher overall rate of foetal complications (67%) compared with similar studies likely reflects differences in inclusion criteria and outcome definitions. Our retrospective design included women with moderate to high disease activity prior to conception and did not exclude severe SLE, contrary to the PROMISSE study (NCT00198068) [12], which enrolled women with inactive or mildly active disease, excluded severe manifestations and did not capture early pregnancy losses, and thus reported lower rates of foetal complications. Maternal complication rates remained low, consistent with our predominantly white cohort, low prevalence of LN and absence of active LN at conception, factors previously associated with a higher risk of flares and pregnancy complications.

Our study has certain limitations, primarily related to its retrospective design. Incomplete serologic data, including C3 and C4, precluded precise calculation of SLEDAI-2K scores for all pregnancies. Nevertheless, disease flares were consistently ascertained through detailed clinical documentation. Information on specific GC dosing and obstetric monitoring was limited; however, GC treatment was classified as continuous or intermittent and interpretations were contextualized with reference to established literature. Importantly, the homogeneity and well-characterized nature of our cohort enhance the internal validity and reliability of our findings.

In summary, our results highlight the importance of preconception counselling, pregnancy planning and adherence to recommended therapies in optimizing pregnancy outcomes among women with SLE. While trends in pregnancy planning and HCQ use have improved, persistently elevated foetal complications in high-risk patients underscore the need for early risk stratification and multidisciplinary care. Future research should refine pregnancy planning definitions and develop interventions to support informed decision-making.

## Supplementary Material

rkaf137_Supplementary_Data

## Data Availability

Data are available upon reasonable request.
