# Supplementary material for: Temporal trends in lupus pregnancy over four decades in a referral centre: pregnancy planning and hydroxychloroquine use are associated with improved outcomes
Source: Rheumatol Adv Pract. 2025 Dec 4;10(1):rkaf137. doi: 10.1093/rap/rkaf137 (PMC12758124; doi:10.1093/rap/rkaf137)
Supplement: rkaf137_Supplementary_Data [file rkaf137_supplementary_data.zip › Supplementary Figure S1.docx]

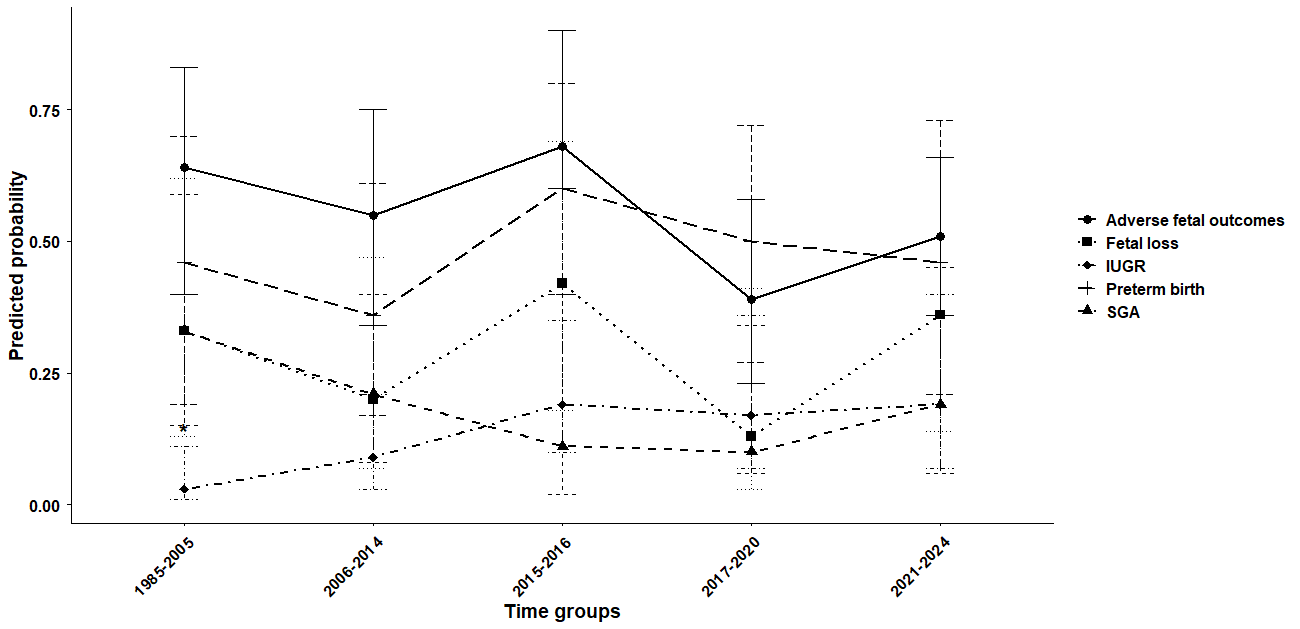


**Supplementary Figure S1. Temporal trends in predicted probabilities of adverse fetal outcomes during lupus pregnancies.**

Predicted probabilities of any adverse fetal outcome, defined as the occurrence of at least one among small for gestational age (SGA), intrauterine growth restriction (IUGR), fetal loss, or preterm birth, and of each individual outcome across five conception year groups (1985-2024) in women with systemic lupus erythematosus (SLE). Estimates were calculated using generalized estimating equations (GEE), and plots show estimated marginal means ± 95% confidence intervals. *p < 0.05 compared to the most recent group (2021-2024).

**Alt text**: Line graph showing predicted probabilities of any adverse fetal outcome, defined as the occurrence of at least one among small for gestational age (SGA), intrauterine growth restriction (IUGR), fetal loss, or preterm birth, and of each individual outcome during pregnancies in women with systemic lupus erythematosus (SLE) between 1985 and 2024. Different black line patterns represent each outcome. The probability of any adverse fetal outcome decreases in the most recent periods, intrauterine growth restriction (IUGR) increases, small for gestational age (SGA) declines and then slightly rises, while fetal loss and preterm birth fluctuate. Significance markers indicate differences from the most recent group.
