# Supplementary material for: Temporal trends in lupus pregnancy over four decades in a referral centre: pregnancy planning and hydroxychloroquine use are associated with improved outcomes
Source: Rheumatol Adv Pract. 2025 Dec 4;10(1):rkaf137. doi: 10.1093/rap/rkaf137 (PMC12758124; doi:10.1093/rap/rkaf137)
Supplement: rkaf137_Supplementary_Data [file rkaf137_supplementary_data.zip › Supplementary Tables Revision.docx]

**Supplementary Table S1** Definitions of adverse fetal outcomes

| **Adverse Fetal Outcomes** | **Definitions** |
| --- | --- |
| Fetal loss |  |
| Early miscarriage | Spontaneous abortion before the 10th week of gestation |
| Late miscarriage | Spontaneous abortion between the 10th and 20th week |
| Stillbirth | Intrauterine fetal demise after the 20th week |
| Neonatal death | Death of a live-born infant within the first 28 days of life |
| Preterm birth | Birth occurring before the completion of the 36th week of gestation |
| Small for gestational age (SGA) | Birthweight below the 10th percentile for gestational age |
| Intrauterine growth restriction (IUGR) | Estimated fetal weight (EFW) or abdominal circumference (AC) below the 10th percentile for gestational age, accompanied by clinical or Doppler signs of abnormal fetal growth or placental insufficiency [18] |

*References correspond to the main manuscript reference list*

**Supplementary Table S2** Definitions of adverse maternal outcomes

| **Adverse Maternal Outcomes** | **Definitions** |
| --- | --- |
| Gestational Hypertension | Blood pressure >140/90 mmHg on two separate occasions at least six hours apart, with onset after the 20th week of gestation, in the absence of a prior history of hypertension, proteinuria, or other signs of preeclampsia [19] |
| Preeclampsia | New-onset hypertension after the 20th week of gestation, accompanied by either proteinuria or signs of end-organ damage [19] |
| Eclampsia | Presence of preeclampsia criteria along with generalized tonic-clonic seizures ("grand mal" seizures) or coma during pregnancy [19] |
| HELLP syndrome | Presence of hemolysis (LDH ≥ 600 IU/L), elevated liver enzymes (AST ≥ 70 IU/L), and thrombocytopenia (platelet count <100,000/μL) occurring during pregnancy or within 7 days postpartum [20] |
| Gestational Diabetes | Glucose intolerance with onset in pregnancy diagnosed through an Oral Glucose Tolerance Test (OGTT) [21] |
| Hyperemesis gravidarum | Severe nausea and vomiting during pregnancy leading to weight loss and electrolyte disturbances [22] |
| Gestational osteoporosis | Decreased bone loss or bone density during pregnancy [23] |
| Oligohydramnios | Decreased amniotic fluid volume for gestational age [24] |
| Polyhydramnios | Abnormal increase in amniotic fluid volume [22] |
| Thrombotic events | Arterial or venous thrombotic events confirmed by the appropriate imaging method |
| Vaginal Bleeding | Any objectively confirmed episode of bleeding through the vaginal canal, irrespective of gestational age and volume (excluding post-partum hemorrhage) |
| Infections | Clinically significant infectious episodes, confirmed by patients reports and medical records review. Only moderate to severe infections (requiring antimicrobial treatment or hospitalization) were included |
| Premature Rupture of Membranes (PROM) | Rupture of membranes before the onset of labor [25] |
| Increased Uterine Artery Resistance | Assessed by Doppler ultrasound |
| Placental abruption | Early separation of the placenta from the uterine wall [26] |
| Disease flare | See definition in main text (Methods) |

*References correspond to the main manuscript reference list*

**Supplementary Table S3** Demographic and clinical characteristics of patients

| Characteristics ^a^ | All patients (n=65) ^b^ |  |
| --- | --- | --- |
| **Age of participants [years, median (IQR)]** | 41 (10.5) | |
| **Age at disease diagnosis [years, median (IQR)]** | 27.5 (14.7) | |
| **SLICC criteria 2012 [n (%)]** |  | |
| Acute cutaneous lupus erythematosus  Chronic cutaneous lupus erythematosus | 48 (73.8)  3 (4.6) | |
| Mucosal Ulcers | 9 (13.8) | |
| Non-scarring Alopecia | 10 (15.4) | |
| Arthritis | 52 (80) | |
| Serositis | 7 (10.8) | |
| Renal disease | 8 (12.3) | |
| Neurologic disease | 3 (4.6) | |
| Hemolytic Anemia | 3 (4.6) | |
| Leukopenia | 14 (21.5) | |
| Thrombocytopenia | 7 (10.8) | |
| ANA | 65 (100) | |
| Anti-dsDNA | 29 (44.6) | |
| Anti-Sm | 7 (10.8) | |
| aPL | 11 (16.9) | |
| Anti-Ro | 19 (29.2) | |
| Hypocomplementaemia | 20 (30.8) | |
| Direct Coombs | 4 (6.2) | |
| **Comorbidities (prior to conception) [n (%)]** |  | |
| Arterial Hypertension | 1 (1.5) | |
| Hypothyroidism | 15 (23.1) | |
| Antiphospholipid syndrome | 7 (10.8) | |
| Fibromyalgia | 3 (4.6) | |
| Sjögren’s syndrome | 3 (4.6) | |

^a^ Clinical characteristics and comorbidities prior to conception are shown. Data were collected retrospectively from medical records. SLICC: Systemic Lupus International Collaborating Clinics, ANA: anti-nuclear antibodies, anti-dsDNA: anti- double stranded DNA, apL: anti-phospholipid antibodies. anti-Sm: anti-Smith ^b^ Continuous variables are presented as median (interquartile range, IQR) and categorical variables as n (%).

**Supplementary Table S4** Temporal trends in pregnancy-related parameters in women with SLE

| **Parameter ^a^** | **β (beta)^b^** | **Odds Ratio (95% CI)^b^** |
| --- | --- | --- |
| **Planned pregnancy** |  |  |
| Group 1 | -2.28 | **0.1 (0.03-0.4)*** |
| Group 2 | -0.92 | 0.4 (0.12-1.38) |
| Group 3 | -1.36 | **0.26 (0.07-0.98)*** |
| Group 4 | -1.29 | **0.27 (0.09-0.87)*** |
| Group 5 (Reference) |  | 1 |
|  |  |  |
| **Preconception counseling** |  |  |
| Group 1 | -0.98 | 0.38(0.06-2.3) |
| Group 2 | -0.98 | 0.38(0.06-2.3) |
| Group 3 | -1.39 | 0.25(0.034-1.82) |
| Group 4 | -0.56 | 0.57(0.1-3.27) |
| Group 5 (Reference) |  | 1 |
|  |  |  |
| **HCQ use** |  |  |
| Group 1 | -0.57 | **0.56 (0.34-0.95)*** |
| Group 2 | -0.32 | 0.73 (0.44-1.2) |
| Group 3 | -0.05 | 0.95 (0.65-1.38) |
| Group 4 | -0.29 | 0.75 (0.51-1.1) |
| Group 5 (Reference) |  | 1 |
|  |  |  |
| **HCQ discontinuation** |  |  |
| Group 1 | 3.5 | **33.2 (11-100)*** |
| Group 2 | 2.86 | **17.5 (6.29-48.6)*** |
| Group 3 | 0.19 | 1.22, (0.49-3) |
| Group 4 | -0.9 | 0.68 (0.6-1.78) |
| Group 5 (Reference) |  | 1 |
|  |  |  |
| **LDA use in not-APS subgroup** |  |  |
| Group 1 | -2.1 | **0.13 (0.03-0.5)*** |
| Group 2 | -0.85 | 0.43 (0.14-1.3) |
| Group 3 | -1.1 | 0.34 (0.11-1.1) |
| Group 4 | -1.03 | **0.36 (0.15-0.87)*** |
| Group 5 (Reference) |  | 1 |
|  |  |  |
| **LDA use in not-APS and with no major organ involvement subgroup** |  |  |
| Group 1 | -2.19 | **0.11(0.03-0.49)*** |
| Group 2 | -0.83 | 0.44 (0.15-1.3) |
| Group 3 | -1.01 | 0.36 (0.12-1.04) |
| Group 4 | -1.16 | **0.32 (0.12-0.70)*** |
| Group 5 (Reference) |  | 1 |
|  |  |  |
| **LWMH use in**  **not-APS subgroup** |  |  |
| Group 1 | -2.23 | 0.11 (0.01-1.38) |
| Group 2 | -0.56 | 0.57 (0.14-2.3) |
| Group 3 | -0.42 | 0.67 (0.16-2.5) |
| Group 4 | -0.68 | 0.51 (0.4-1.82) |
| Group 5 (Reference) |  | 1 |
|  |  |  |
| **LMWH use in not-APS and no major organ involvement subgroup** |  |  |
| Group 1 | -2.44 | **0.09(0.02-0.39)*** |
| Group 2 | -0.54 | 0.56 (0.18-1.94) |
| Group 3 | -0.49 | 0.62 (0.23-1.73) |
| Group 4 | -0.89 | 0.4 (0.15-1.15) |
| Group 5 (Reference) |  | 1 |
|  |  |  |
| **GC use for non-flare indications** |  |  |
| Group 1 | 1.98 | **7.22 (1.1-50)*** |
| Group 2 | 1.53 | 4.6 (0.42-22.5) |
| Group 3 | 1.12 | 3 (0.42-22.5) |
| Group 4 | 1.5 | 1.5 (0.16-14.6) |
| Group 5 (Reference) |  | 1 |
|  |  |  |
| **Continuous GC use during pregnancy** |  |  |
| Group 1 | 2.24 | **9.36 (1.25-65)*** |
| Group 2 | 1.29 | 3.63 (0.74-17.7) |
| Group 3 | 1.34 | 3.80 (0.63-23.1) |
| Group 4 | -0.003 | 0.99 (0.29-3.47) |
| Group 5 (Reference) |  | 1 |
|  |  |  |
| **Adverse fetal outcome** |  |  |
| Group 1 | 0.55 | 1.73 (0.54-5.54) |
| Group 2 | 0.17 | 1.19 (0.42-3.34) |
| Group 3 | 1.1 | 3 (0.8-9.7) |
| Group 4 | -0.49 | 0.61 (0.31-1.29) |
| Group 5 (Reference) |  | 1 |
|  |  |  |
| **Multiple Adverse fetal outcomes** |  |  |
| Group 1 | -0.62 | 0.54 (0.08-3.64) |
| Group 2 | -2.52 | 0.59 (0.1-3.39) |
| Group 3 | 0.68 | 1.56 (0..48-8.12) |
| Group 4 | -0.63 | 0.53 (0.4-2.03) |
| Group 5 (Reference) |  | 1 |

Temporal trends in pregnancy-related parameters across five time groups, with Group 5 (most recent) as the reference. SLE: Systemic Lupus Erythematosus, HCQ, hydroxychloroquine LDA: low-dose aspirin, LMWH: low-molecular-weight heparin, APS: antiphospholipid syndrome, GC: glucocorticoids. Group 1: 1985-2005; Group 2: 2006-2014; Group 3: 2015-2016; Group 4: 2017-2020; Group 5: 2021-2024. **^b^** Analyses were performed using Generalized Estimating Equations (GEE). Results are reported as β coefficients, and odds ratio (OR) with 95% Confidence Intervals (CI). *Statistically significant associations (p<0.05).

**Supplementary Table S5** Temporal trends in patients’ and disease characteristics

| **Parameter ^a^** | **β (beta)^b^** | **Odds Ratio (95% CI)^b^** | **Estimated Marginal Means (95% CI)^b^** |
| --- | --- | --- | --- |
| **Maternal age at conception (years)** |  |  |  |
| Group 1 | -7.9 | **0.012 (0.01-0.03)*** | 27.89 (26.17-29.62) |
| Group 2 | -4.1 | **0.016 (0.01-0.08)*** | 31.67 (30.27-33.08) |
| Group 3 | -3.9 | **0.02 (0.01-0.13)*** | 31.88 (29.74-34.02) |
| Group 4 | -2.1 | **0.12 (0.05-0.3)*** | 33.67 (32.3-35.03) |
| Group 5 (Reference) |  | 1 | 35.78 (34.4-37.15) |
|  |  |  |  |
| **Smoking at the time of conception** |  |  |  |
| Group 1 | 0.083 | 1.09 (0.53-2.2) | 0.3 (0.16-0.49) |
| Group 2 | 0.29 | 0.74 (0.38-1.44) | 0.23 (0.12-0.4) |
| Group 3 | 0.06 | 1.06 (0.82-1.38) | 0.3 (0.19-0.43) |
| Group 4 | 0.07 | 1.07 (0.97-1.18) | 0.3(0.2-0.43) |
| Group 5 (Reference) |  | 1 | 0.29 (0.18-0.42) |
|  |  |  |  |
| **History of major organ involvement** |  |  |  |
| Group 1 | -2.45 | 0.09 (0.06-1.19) | 0.04 (0.01-0.34) |
| Group 2 | -0.31 | 0.73 (0.24-2.27) | 0.26 (0.12-0.47) |
| Group 3 | -0.79 | 0.92 (0.32-2.66) | 0.31 (0.16-0.52) |
| Group 4 | 0.09 | 1.1 (0.47-2.56) | 0.34 (0.19-0.53) |
| Group 5 (Reference) |  | 1 | 0.32 (0.18-0.52) |
|  |  |  |  |
| **History of LN** |  |  |  |
| Group 1 | -5.9 | **0.003(0.001-0.01)*** | 0.02(0.01-0.9) |
| Group 2 | -0.41 | 0.66 (0.19-2.35) | 0.27(0.13-0.49) |
| Group 3 | -0.39 | 0.68 (0.19-2.45) | 0.28 (0.13-0.5) |
| Group 4 | -0.24 | 0.79 (0.23-2.7) | 0.31 (0.15-0.53) |
| Group 5 (Reference) |  | 1 | 0.36 (0.19-0.57) |
|  |  |  |  |
| **History of any complications in previous pregnancy** |  |  |  |
| Group 1 | -0.79 | 0.45 (0.14-1.53) | 0.35 (0.18-0.57) |
| Group 2 | -1.39 | **0.25 (0.07-0.89)*** | 0.23 (0.1-0.44) |
| Group 3 | -0.25 | 0.78 (0.24-2.51) | 0.40 (0.28-0.60) |
| Group 4 | -0.92 | 0.13 (0.12-1.32) | 0.32 (0.16-0.53) |
| Group 5 (Reference) |  | 1 | 0.54 (0.35-0.72) |

**^a^** Temporal trends in patients’ and disease characteristics across five time groups, with Group 5 (most recent) as the reference. Group 1: 1985-2005; Group 2: 2006-2014; Group 3: 2015-2016; Group 4: 2017-2020; Group 5: 2021-2024. LN: lupus nephritis.

**^b^** Analyses were performed using Generalized Estimating Equations (GEE). Results are reported as β coefficients, odds ratio (OR) with 95% Confidence Intervals (CI) and Estimated Marginal Means represent with 95% CI.

*Statistically significant associations (p<0.05).
